# Free-Standing Graphene Oxide and Carbon Nanotube Hybrid Papers with Enhanced Electrical and Mechanical Performance and Their Synergy in Polymer Laminates

**DOI:** 10.3390/ijms21228585

**Published:** 2020-11-14

**Authors:** Manoj Tripathi, Luca Valentini, Yuanyang Rong, Silvia Bittolo Bon, Maria F. Pantano, Giorgio Speranza, Roberto Guarino, David Novel, Erica Iacob, Wei Liu, Victor Micheli, Alan B. Dalton, Nicola M. Pugno

**Affiliations:** 1 Department of Mathematics and Physical Sciences, University of Sussex, Brighton BN1 9QH, UK; tracy.rong0202@gmail.com (Y.R.); A.B.Dalton@sussex.ac.uk (A.B.D.); 2 Department of Civil and Environmental Engineering, University of Perugia and INSTM Research Unit, Strada di Pentima 4, 05100 Terni, Italy; luca.valentini@unipg.it (L.V.); silvia.bittolobon@unipg.it (S.B.B.); 3 Laboratory of Bio-Inspired, Bionic, Nano, Meta Materials & Mechanics, Department of Civil, Environmental and Mechanical Engineering, University of Trento, via Mesiano 77, 38123 Trento, Italy; maria.pantano@unitn.it (M.F.P.); roberto.guarino@alumni.unitn.it (R.G.); ddsnovel@gmail.com (D.N.); 4 Centre for Materials and Microsystems, Fondazione Bruno Kessler, via Sommarive 18, 38123 Trento, Italy; g.speranza@fbk.eu (G.S.); iacob@fbk.eu (E.I.); liuwei4176@hotmail.com (W.L.); micheli@fbk.eu (V.M.); 5 Department of Industrial Engineering, University of Trento, via Sommarive 9, 38123 Trento, Italy; 6 Istituto di Fotonica e Nanotecnologie, IFN-CNR, via alla Cascata 56/C, 38123 Trento, Italy; 7 School of Engineering and Materials Science, Queen Mary University of London, Mile End Road, London E1 4NS, UK

**Keywords:** nanotechnology, carbon nanotubes, electrical properties, mechanical properties, composites

## Abstract

Hybrid nanomaterials fabricated by the heterogeneous integration of 1D (carbon nanotubes) and 2D (graphene oxide) nanomaterials showed synergy in electrical and mechanical properties. Here, we reported the infiltration of carboxylic functionalized single-walled carbon nanotubes (C-SWNT) into free-standing graphene oxide (GO) paper for better electrical and mechanical properties than native GO. The stacking arrangement of GO sheets and its alteration in the presence of C-SWNT were comprehensively explored through scanning electron microscopy, X-ray photoelectron spectroscopy (XPS) and X-ray diffraction. The C-SWNTs bridges between different GO sheets produce a pathway for the flow of electrical charges and provide a tougher hybrid system. The nanoscopic surface potential map reveals a higher work function of the individual functionalised SWNTs than surrounded GO sheets showing efficient charge exchange. We observed the enhanced conductivity up to 50 times and capacitance up to 3.5 times of the hybrid structure than the GO-paper. The laminate of polystyrene composites provided higher elastic modulus and mechanical strength when hybrid paper is used, thus paving the way for the exploitation of hybrid filler formulation in designing polymer composites.

## 1. Introduction

Graphene offers numerous attributes to enhance the device performance, but its limited chemical interaction with most materials highlighted the importance of its functional derivative, GO. The cost-effective GO-precursor and ability to yield at tons scale make GO a premium choice for the fabrication of graphene-based devices [1,2]. The structure of GO possesses a graphene-based lattice along with various oxygen-containing epoxy and a hydroxyl group at the basal plane and carbonyl at the edges [3]. These functional groups lead to dispersion in solvents, anchoring sites for active species, strengthening fillers-matrix interfaces through covalent bonds [4]. Moreover, GO powders can also be transitioned into the free-standing paper/foil-like formation with up to a few micron thicknesses, referred to as “GO-paper” [5]. Unlike the GO-powdered configuration, GO-paper has exceeded its stability, especially in wet and ionic environments [6], which distinguishes its hydration and solvation characteristics [7] from its predecessor. The stability of GO-paper is achieved through the crosslinking of GO sheets, especially in the presence of multivalent cations [8]. The GO-paper produced by the simple process of filtration and drying shows higher mechanical properties (e.g., elastic modulus ~32 GPa and fracture strength ~ 132 MPa) as compared to the traditional carbon- and clay-based paper [9,10]. Thus, free-standing GO-paper has gained widespread applications in impermeability to fluids [11], structural composites [12] and optoelectronics components [13].

Nevertheless, GO sheets also show some limitations to practical applications. The presence of functional groups induces structural and ionic defects that alter their properties. Several challenges, such as restacking and multilayer thickening of GO, significantly affects electrical conductivity with higher resistivity [14]. Moreover, the presence of oxygen-based functional groups induces electrical insulation in GO of band gap ≈ 3.7 eV [15,16]. Although chemical and thermal treatments can improve the conductivity by reduction (rGO), the inherent characteristics of GO due to the presence of functional groups would also be compromised. The residual defects and vacant sites after removal of oxygen groups limit the electronic quality of the reduced graphene [17]. To widen the application of GO and to preserve its intrinsic solubility and processability, carbon nanotubes can be taken into account due to their synergy observed in electronic, thermal and mechanical properties [18,19,20,21,22]. Carbon nanotubes can effectively prevent GO from restacking even without binders and also reinforce its electrical conductivity and ion diffusion [21,22]. In turn, GO sheets also prevent the nanotube agglomeration by suppressing the van der Waal forces and intermolecular interactions through electrostatic and steric repulsive forces. It has been observed that the π-π bond interaction between GO and carbon nanotubes allows good dispersion of the nanotubes in aqueous medium [20] and inhibits their aggregation [23], which might play a crucial role for the reproducible results. The hybrid structure produced by encapsulation of carbon nanotubes between negatively-charged GO is used to fabricate high dielectric performance devices [20]. The intercalated carbon nanotubes have created the hybrid system for charge storage applications [21,24]. A recent application in this field is the use of GO/carbon nanotube composite materials as electrodes in batteries [25,26] and in supercapacitors [27], showing promising performance in ion transport and of storage capacity, respectively. Furthermore, the mechanical properties of nanocomposites with GO/carbon nanotube fillers have been recently investigated, showing increased tensile strength [28,29]. Nevertheless, rare research focused on the surface chemistry and morphology of hybrid architectures from the bench to composite applications.

In this study, we prepared a hybrid composite membrane by the integration of carboxylic functionalized SWNT (C) with GO sheets. The presence of GO sheets minimises aggregation of C-SWNT, while the elongated C-SWNT acts as a bridge between different GO sheets. Mechanical and electrical properties along with capacitance ability of the GO-paper and GO/C-SWNT hybrid paper were studied from the nano to the microscale. It resulted that C-SWNT incorporation in GO sheets increased the electrical conductivity through p-type doping, and enhanced the mechanical properties of the hybrid composite better than individual GO-paper. The integration of GO and C-SWNT results differed in thermal behaviour, surface chemistry and crystallography than native GO sheets. Later, the hybrid membrane (as a filler) is used to fabricate polystyrene (PS) laminates. We found a synergy of both nano-carbons in the mechanical properties of polymer matrix laminate. Our combined experiments at the nano and microscale confirm that the addition of SWNTs provides useful background information which allows the design of laminated composites with improved thermal decomposition, mechanical and electrical properties.

## 2. Results and Discussion

### 2.1. Morphology of GO and GO/C-SWNT

**Figure 1:**
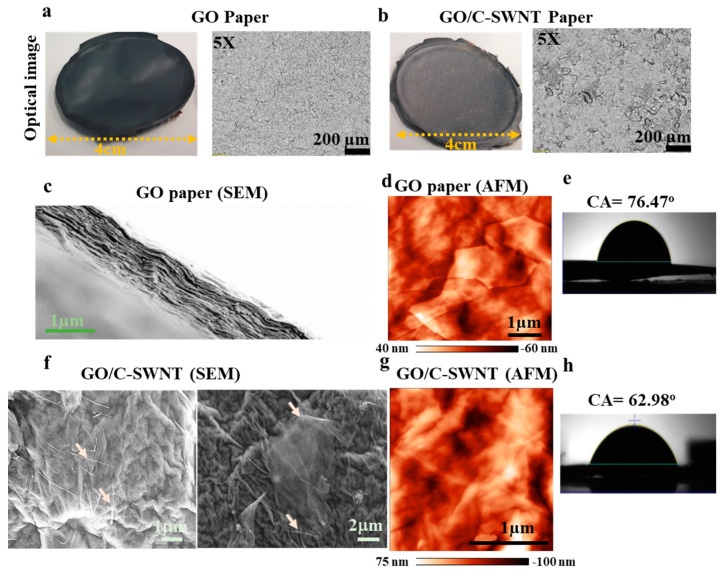
(**a**) Photograph (4 cm) and optical image resolution (5×) for free-standing GO and (**b**) of GO/C-SWNT paper. (**c**) SEM image (the background has been whitened, for clarity) of a GO-paper showing packing of each GO layer conglomeration. (**d**) AFM image of individual GO sheets cross-stacked to produce a GO-paper. (**e**) A water drop profile on a GO sheet with a contact angle of 76.47° over GO sheets. (**f**) SEM image of a GO/C-SWNT paper shows entangled CNTs network; inset reveals an area of the cross-section. (**g**) AFM image of GO/C-SWNT paper shows disruption of GO sheet arrangement in vertical stacking, some C-SWNT are shown by marked arrows. (**h**) The water contact angle measurement of the GO/C-SWNT paper is 62.98° showing higher hydrophilic characteristics than GO-paper. The “+” sign indicates the focused location of the objective lens at the topmost curvature of water droplet.

Free-standing GO-paper with thickness 10 ± 2 µm was produced by filtration followed by drying an aqueous dispersion of graphene oxide sheets. The GO sheets are relatively more shiny than their C-SWNT hybrid due to the difference in the functional group composition at the surface of GO and hybrid paper [30]. In the resolved optical image of 5× resolution, several craters and microscopic surface cracks have been observed in the hybrid, Figure 1a,b. It has been reported that GO-paper thicker than 5 micron mostly reflects a blackish color in white light [31]. The incorporation of the nanotubes disrupts the stacking arrangement of the GO sheets during drying and induces disorder in the stacking arrangement. The packing of each layer sandwiched between fewer densely stacked arrangements is revealed by an SEM micrograph at the cross-section area (Figure 1c). The high resolution of the SEM images reported in the inset of Figure 1c reveals the “waviness” of each skin layer bigger than 1 µm, which is responsible for the overall thickness and surface roughness of the paper. Evaporation causes drying of the upper surface of the hydrosol, which in turn increases the concentration of GO sheets by squeezing water molecules out from the GO matrix and leads to self-assembled sheets. The immobilized water molecules persisted in the GO matrix by hydrogen bond through acceptor and donor sites on neighbouring graphene oxide sheets [32].

Atomic force microscopy (AFM) images carried out using a sharp tip apex (radius ≈ 10 nm) revealed smoother GO sheets of roughness (rms, root mean square) equal to 14 nm (see Figure 1d). The wetting characteristics of the GO-paper measured through a drop shape contact angle measurement is around 76.5°, which represents its hydrophilic surface and its hybrid structure (whose contact angle is ≈ 63°). The addition of a functional group (-COOH) from the SWNTs and the higher number of crater-like shapes at the surface which exposes edges may be responsible for the higher hydrophilic surface [3]. The incorporation of C-SWNT into GO-paper leads to higher roughness (Figure 1f,g) that indicates a complex mixture of nanotubes and intercalated water molecules [18]. Moreover, due to the longer length of C-SWNT than the lateral size of GO sheets, the CNTs are shared by neighbouring GO sheets and act as a bridging site that might influence the mechanical properties. The C-SWNT altered the packing of GO sheets and their interconnectivity at the nanoscale, which might be the reason for the higher roughness (rms = 29 nm).

### 2.2. Crystallographic and Chemical Analysis

**Figure 2:**
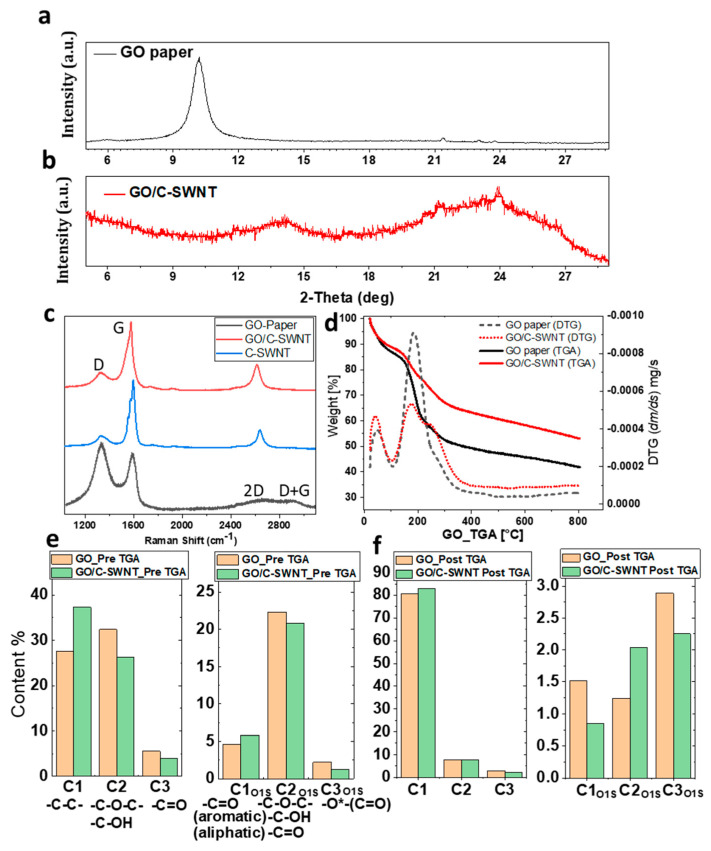
(**a**,**b**) XRD spectra of GO and GO/C-SWNT paper for 2θ range between 5–30°. (**c**) Raman spectra of pure C-SWNT, GO-paper and GO/C-SWNT in the range from 1000 cm^−1^ to 3200 cm^−1^ showing characteristics D, G, and 2D peaks. Inset shows the zoomed region of 2D and D+G peaks for the native GO-paper. (**d**) TGA (thermogravimetric analysis) recorded from 20 °C to 800 °C with the degradation of GO and GO/C-SWNT in Ar- atmosphere, inset DTG curves shows the rate of mass loss at different temperatures. (**e**,**f**) Histogram from XPS data measured for C1S and O1S of GO and GO/C-SWNT of pre and post TGA treatment showing the presence of aliphatic and functional carbon groups.

XRD spectra validate the amorphization-induced roughness of the hybrid system. The XRD spectra of a GO-paper showing a peak at 2θ = 10.1° for 002 planes indicates the arrangement of individual GO sheets in their crystal lattice, for *d*-spacing 0.8 nm (Figure 2a). Addition of C-SWNT leads to broad peaks at 2θ = 14.2° and 24° (Figure 2b) which reveal the interaction between the functionalized C-SWNT and GO sheets followed by the reduction [33]. The vanishing of the GO peak after C-SWNT addition indicates that the inclusion of nanotubes hinders the ordered stacking of GO sheets along the c-axis [34] with slightly lower *d*-spacing of nearly 0.6 nm.

Raman spectra of GO-paper shows prominent peaks of D (~1335 cm^−1^) and G (~1588 cm^−1^), which correspond to sp^3^ and sp^2^ hybridised carbon states, respectively. The D peak appears due to the disorder and imperfection of the carbon crystallites and the G peak is assigned to one of the two E_2g_ modes during stretching vibration of graphitic carbon atoms [35]. There is a significant increase in sp^2^ hybrid carbon after addition of C-SWNT as illustrated by G peak, see the spectra GO/C-SWNT in Figure 2c. The I_G_/I_D_ variation from C-SWNT (5.3 ± 0.3), GO/C-SWNT (3 ± 0.4) and GO (0.8 ± 0.03) paper shows lower sp^2^ carbon atoms in GO-paper and the intermediate in the hybrid. The intensity of the 2D peak (~2650 cm^−1^) in GO-paper is extremely low (inset Figure 2c), which is enhanced by a factor ~5 after addition of C-SWNT in GO-paper using normalised G peak values. The broader FWHM (~68 cm^−1^) of the 2D peak in GO/C-SWNT than the pristine C-SWNT (~53 cm^−1^) show the contribution from both nanotubes and GO [36]. The D+G bump is activated by the presence of defects in the GO-paper [37]. There is a significant red shift in the 2D peak (≈36 cm^−1^) and a slight red shift observed in the G peak position ≈8 cm^−1^ in the GO-C-SWNT sample as compared to C-SWNT, which shows the combination of the compressive strain and doping in the C-SWNT [35]. The presence of oxidized sheets in GO around CNTs act as an electron-withdrawing group and leads to p-type doping of the native carbon materials (CNT, graphene, etc.). The charge exchange between sp^2^ carbon atoms and the oxidative functional group in GO-sheets induces stable and strong p-doping in the C-SWNT network useful for charge storage [38]. We have demonstrated the local p-doping in the C-SWNT due to the surrounded GO by Kelvin Probe Force Microscope (KPFM) analysis in the subsequent section.

The thermal induced degradation of GO-paper and the GO/C-SWNT hybrid system was investigated employing TGA, as shown in Figure 2d. In the temperature range between 20 and 120 °C weight losses of ≈15.5% for GO-paper and 12.5% for the GO/C-SWNT paper were observed. It corresponds to the removal of adsorbed water molecules and labile oxygenic functional groups [18]. The subsequent increase of the temperature up to 250 °C resulted in further mass reductions of ≈30 wt% (GO-paper) and 20 wt% (GO/C-SWNT). It depicts thermal-induced reduction of the samples that leads to the decomposition and causes the emission of CO and CO_2_ gases. Furthermore, high temperature leads to a gradual loss of mass due to the removal of residual oxygen by the continuous evolution of CO and CO_2_ gases as generated in the second step [39,40]. The derivative thermogravimetric (DTG) plots in Figure 2d, reveal the rate of mass loss at different stages of temperature. The peak area increased in the DTA curves corresponding to the decomposition reactions for each of the constituent molecules by progressive overlapping of peaks with increasing temperature. The higher rate of mass dropped appears in the range of 170–250 °C, which is lower for the hybrid sheet. The total mass loss in GO/C-SWNT paper at 800 °C is 47%, which is lower than that observed for pristine GO-paper (wt% 60). The drop in mass from SWNT is up to 26% in the entire temperature (see Supplementary Material Figure S3), which indicates the majority of mass loss from the GO sheets. The mass loss in GO and the GO/C-SWNT paper is associated with a reduction of attached functional groups (particularly oxygenic) with the increasing temperature. The presence of a higher amount of trapped water molecules in GO-paper is responsible for the formation of CO and CO_2_ and their creation requires the breaking of C–C bonds in the GO lattice [41]. Therefore, the water molecules can significantly influence the thermal stability of GO-paper. Kavinkumar et al. [42] pointed out that the formation of the π-π stacking interaction at the basal plane of aromatic GO sheets with sidewalls of CNTs leads such behavior in the hybrid structure rather than native GO-paper.

XPS was performed for chemical identification of the samples at two different stages of pre- and post-treated TGA. At the pre-treated stage, the chemical information of synthesised GO and GO/C-SWNT paper reveals C-SWNT addition that leads to a drop in -C-O-C- and -C-OH functional groups from 32.6% to 26.2% (Figure 2e). It reflects that the addition of the C-SWNT also influences the basal plane of GO sheets, which is rich in epoxy and hydroxyl groups. The XPS spectra are given in Supplementary Information Figure S4, Tables S1 and S5, Table S2 respectively. The removal of hydroxyl and epoxy groups occurred during the drying of the hybrid paper. Recently, Núñez et al. [18] observed a similar effect of displacement of water molecules after the addition of 10 to 15 wt% of carboxylic carbon nanotubes. Several mechanisms drive the process of drying of GO dispersion followed by a complex procedure of self-alignment of GO sheets. These may include: gravitational force in low viscous media [43], steric hindrance [44], π-π interaction and van der Waals forces consisting of both hydrophilic edges due to ionisable carbonyl/carboxyl groups and hydrophobic basal planes with isolated epoxy/hydroxyl groups [45]. It has been proved that a higher amount of carboxyl functional groups with lower C/O ratios leads to unmanageable alignment [46], which appeared after the addition of C-SWNT responsible for amorphization of the hybrid structure. It explains that a hydrophilic character (from contact angle measurement) of the GO/C-SWNT is due to the presence of a carboxylic group from the C-SWNT and edges of disordered regions.

XPS analysis of post-treated TGA allows us to compare the loss of the functional group between GO and the hybrid paper (see Supplementary Material Figure S6, Tables S3 and S7, Table S4). The carbon to oxygen ratio (C/O) for the GO and GO/C-SWNT paper are measured as 2.33 and 2.48 respectively, and increases up to 15.3 and 17.1 after the TGA treatment. Typically, the GO flakes have a C/O ratio from 1.5 to 2.5 but it varies with the choice of production process [47]. This C/O ratio influences the surface chemistry to mechanical properties of the polymer composite [48]. The removal of oxygen functional groups during TGA in GO-paper by deconvolution of C1s components are as follows: -C-O-C, C-OH from 32.3% to 8%, C=O from 5.5% to 2.8% and -O-(C=O) from 1.7% to 1.3%. The GO/C-SWNT paper has shown the thermal decomposition of functional groups such as -C-O-C-, C-OH from 26% to 8%, C=O from 4% to 2.2% and -O-(C=O) from 1.3% to 0.6%. The O1S components are also showing a drop in the oxygenic functional groups for both GO and GO/C-SWNT paper after TGA treatment.

### 2.3. Electrical and Mechanical Analysis

**Figure 3:**
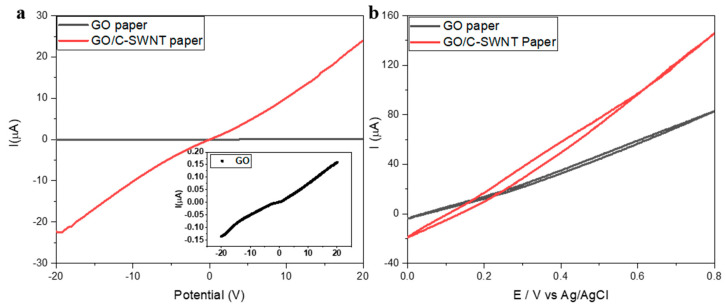
(**a**) I-V curve of GO-paper and GO/C-SWNT paper between −20 to 20 V. Inset shows resolved I-V curve from GO-paper. (**b**) Cyclic voltammetry for GO and GO/C-SWNT paper in buffer solution (PBS = 7).

**Figure 4:**
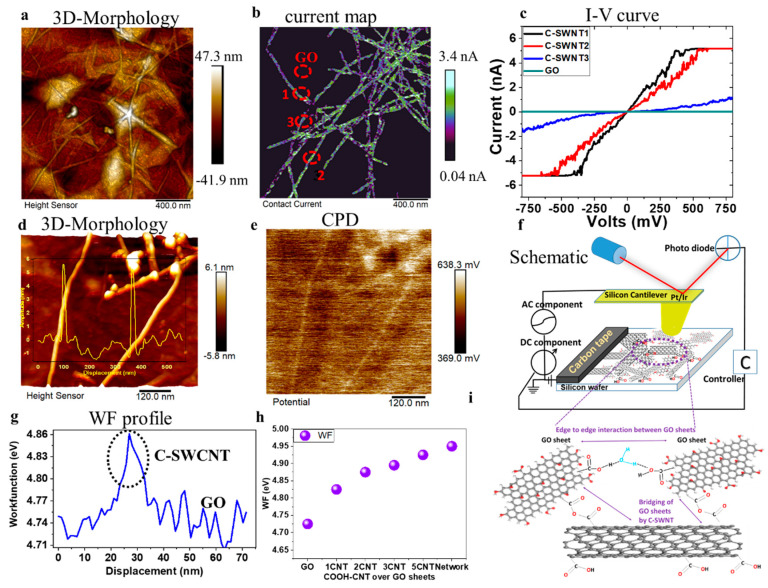
(**a**) 3D-AFM topography of C-SWNTs on GO surface, showing their distribution and networking with each other over GO sheets. (**b**) The local conductivity map contrast between C-SWNT and GO sheets under a bias voltage of 900mV. (**c**) I-V curve carried out at 3 different C-SWNTs marked as 1, 2 and 3 showing the impact of functional groups on local electrical property. (**d**) 3-D morphology carried under KPFM operation in first-pass scanning shows individual C-SWNTs of a diameter nearly 5nm connected to its network. (**e**) The CPD (mV) contrast (second pass) between individual C-SWNT illustrating the difference in the work function (WF) between GO sheets, individual C-SWNT and its network. (**f**) Schematic view of the AFM set-up of the same sample used for C-AFM and KPFM measurements. (**g**) WF profile of an individual C-SWNT marked by dotted circle which is higher than in the surrounded GO. (**h**) The trend of increasing WF values with accumulation of C-SWNTs. (**i**) Enlarged representation of the proposed bridging mechanism of GO sheets by C-SWNT and the edge interaction between GO sheets through water molecules [46,57].

The bridging of the C-SWNT in GO/C-SWNT structure also improves the electrical properties. Current-voltage characteristics of GO-paper and GO/C-SWNT are shown in Figure 3a. The addition of C-SWNT enhances the conductivity in the hybrid paper with respect to the GO system (inset Figure 3a). Specifically, the conductivity increases from 1.2 × 10^−6^ S/m for the GO-paper to 6.5 × 10^−5^ S/m for the hybrid GO/C-SWNT paper. The surface conductivity depends on the conductive percolated network established by C-SWNTs and the stacking arrangement of GO sheets in the hybrid paper [49]. Tang et al. [19] observed that a lower concentration of CNT in GO sheets could constrain the full length of flexible CNTs, thus not resulting in a close-packed arrangement to form effective pathways in the paper. A higher weight fraction of CNT (>15%) promotes the mobility due to the formation of a percolated network in GO sheets at the entire surface through bridging as illustrated in Figure 4. The cyclic voltammetry (CV) results show that the C-SWNT in GO-paper provides a larger capacitance as compared with the pure Ohmic behaviour of native GO. The charge under the CV curve increases to 35 µC for GO/C-SWNT, while for GO it is measured as 10 µC. The presence of CNTs promotes the electrochemical conversion of the GO into its reduction through a controlled electron-transfer process. It leads to the higher current flow in the electrochemical cell and the electrochemical performance better than individual GO-paper [50].

The conductivity of the C-SWNT distribution at the nanoscale is illustrated in Figure 4a,b which shows the percolating pathway of CNTs. The GO sheets (as an insulator) and the isolated nanotubes, which are not connected to the network, are showing minimal current signals. Thus, the measured current values in the C-SWNTs network is from the electrode (carbon tape) connected to the sample as shown in the schematic Figure 4f,g. The carboxylic groups attached to CNTs affect the intensity of the current by a factor of 1.5–2 and appear as dark color patches (i.e., lower values of current). The non-zero values of the current at the carboxylic group is due to the larger apex area of the conductive tip than the functional group, which might cause a tunneling of the current [51]. The effect of functionalization is also reflected in the I-V curve plots of three different individual C-SWNTs marked as number 1 (non-ohmic > 140 mV and < −140 mV), 2 (ohmic) and 3 (lower current intensity) in Figure 4c. This is due to the defective sites generated by the presence of -COOH groups causing a redox reaction at a higher magnitude of applied voltage. Nevertheless, the intensity of the current values of CNT under the applied voltage range (−500 to 500 mV) is significantly higher than that of the GO sheet.

KPFM provides concurrent information of the sample topography, and its local contact potential difference (CPD, mV), down to a lateral resolution of 5–50 nm. The CPD is the contact potential difference between the tip and the sample associated with local work function (*ϕ*) and is defined as

V_CPD_ = (*φ_tip_* − *φ_sample_*)/(−*e*) (1)

*e* being the elementary charge [52,53]. The local CPD is the potential of the GO/C-SWNT with respect to the tip apex. Therefore, in the absence of isolated charges and dipoles, CPD values represent the difference between the sample and the tip work function [54]. The presence of C-SWNT modifies the CPD between the tip and the GO surface as reported in Figure 4d-h. The exchange of charge between GO sheets and the higher number of sp^2^ carbon atoms in C-SWNT are responsible for the potential variation. Figure 4d,e clearly illustrates topography and the CPD map of two individual C-SWNT. The work function profile from an individual C-SWNT is measured in Figure 4g, which is higher than the surrounded GO sheet. The accumulation of the C-SWNTs into the network enhances their CPD output as compared to that of the individual C-SWNT (Figure 4e,h). There is a systematic increase in the average values of the work function (eV) with the accumulation of C-SWNTs.

The arrangement of the AFM set-up for electrical and potential measurements is shown through a schematic view at Figure 4f. In this set-up, lower CPD values represent lower work function of the material, suggesting that GO has the lowest work function (4.72 eV) and the C-SWCNT network has the highest (4.92 eV), suggesting p-type doping in the later. The intermediate value of an individual C-SWCNT (4.88 eV) is due to weak CPD signals measured from the single carbon nanotubes, which gradually increases with accumulation. It is to be noted that material topography, substrate interaction and local environment significantly influences the local CPD values, and consequently the work function [53]. Our KPFM measurements were carried out in an ambient atmosphere at 33% humidity. Therefore, the presence of water molecules over the sample surface cannot be neglected. These results are in good agreement with the work carried out by Ago and coworkers (4.4 eV for air oxidised SWNT and 4.8 eV for plasma oxidised SWCNT) [55]. A wide variation of the work function is reported for GO with values ranging from 3.7 to 5.1 eV depending on the different functional groups; nevertheless, the most used value is nearly 4.7 eV [56]. The larger distance between the vacuum and the Fermi energy levels is responsible for the increase in the work function revealing the electron binding ability of a material [53]. Thus, the C-SWCNT gets p-type doping and acts as a donor material to the surrounded GO and the electrical performance is achieved through hole mobility.

**Figure 5:**
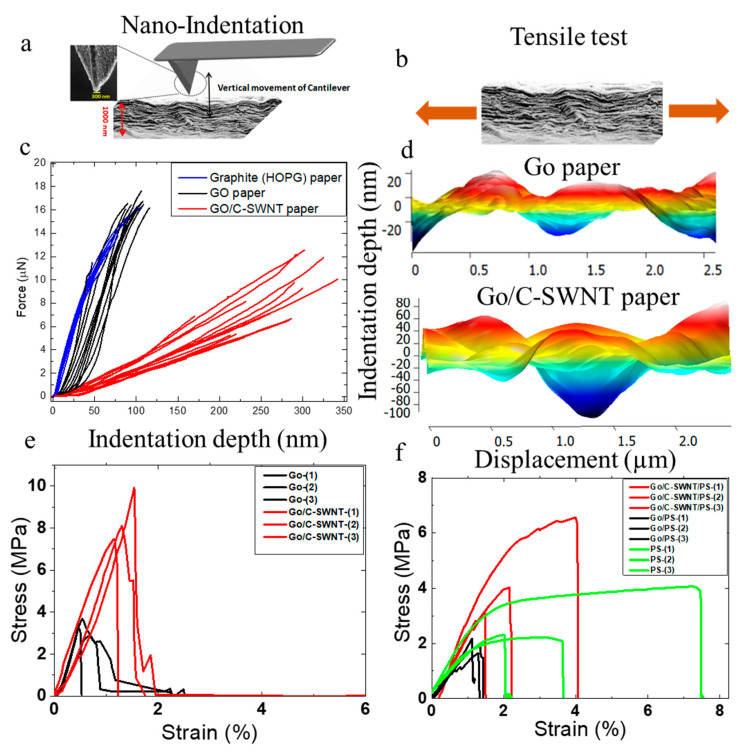
(**a**) SEM image of the AFM tip apex and the cross-section region of GO-paper. (**b**) A schematic view showing a uniaxial tensile force applied to a GO-paper. (**c**) Force-penetration plots recorded for a GO-paper, GO/C-SWNT and Graphite (HOPG, reference sample) using a DLC-coated silicon tip (inset SEM image probe apex). The data dispersion is associated with the degree of uniformity in the packing of individual layers as well as the roughness of the topmost layers. (**d**) The indentation depth measured after force-distance spectroscopy shows higher depth (nm) in hybrid GO/C-SWNT. (**e**) Stress-strain curves obtained from tensile tests on GO-paper and GO/C-SWNT. (**f**) Stress-strain curve for PS laminates obtained from GO and GO/C-SWNT fillers.

The mechanical properties of GO and GO/C-SWNT papers were investigated through AFM-nanoindentation and tensile tests. Figure 5a,b illustrate the schematic arrangements of the test conditions in which a diamond-like carbon (DLC) coated silicon tip was used for nanoindentation and a uniaxial force was applied to induce strain in the samples, respectively. Figure 5c shows the force-penetration curves recorded for GO-paper, GO/C-SWNT and the reference sample (HOPG, graphite) using a triangular pyramidal tip (see Supplementary Material Figure S8 for the tip profile). The force-depth curves are obtained by following the procedure described by Annamalai et al. [58] The analytical solution using a sharp indentation of an elastic substrate was provided by Sneddon [59], while Sirghi et al. [60] extended it to the nanoindentation by a square pyramidal AFM tip. Here, we derive the Young’s modulus and the adhesion energy of the substrate by investigating the tip-sample contact area and the projected area implemented by considering the triangular geometry of the tip apex. Table 1 shows the results of the best fits according to the following equation:

(2) $$F=\frac{2}{\pi }\sqrt{\frac{3\sqrt{3}}{\pi }}\;\frac{E}{1-\nu^{2}}\;\tan\alpha \;h^{2}-\gamma \frac{24\sqrt{3}}{\pi^{2}}\;\frac{\tan\alpha }{\cos\alpha }\;h$$

where *F*, *h*, *γ*, *α*, *E* and *ν* are the total force (including adhesion), indentation depth, adhesion energy, tip apex conical angle, Young’s modulus and Poisson’s ratio, respectively. The details of the obtained equation are given in the Supplementary Material at Supplementary Information 1 (analysis of the nanoindentation curves), where we have adapted the results provided in Reference [54] to our tip geometry. The examples of the fit using Equation (2) for GO-paper, GO/C-SWNT and HOPG are given in Supplementary Material Figure S9.

The *E* extracted from nanoindentations for GO/C-SWNT paper is up to 1/6 times smaller than GO-paper. It reflects the lower stiffness of the hybrid paper in an out-of-plane direction. The amorphous nature of GO/C-SWNT comprising structural disorder causes an indentation depth up to four times higher than GO-paper under a similar applied force (Figure 5d). It seems that the local stress induced from the tip apex cannot be transferred to the nanotubes in the out-of-plane direction. GO-paper has smaller interlayer separation and the intercalation of water molecules forms hydrogen bonds with other GO sheets in an out-of-plane direction. Furthermore, an organized stacking arrangement of GO sheets distributes the normal stress induced by the tip apex. Our argument is further validated by using HOPG as a reference that represents a higher crystalline order of carbon as a free-standing paper of minimal interlayer separation of 0.35 nm. Thus, the highest stiffness values are observed in the HOPG as compared to GO and GO/C-SWNT paper. Several indentation measurements up to 15 times at each sample for different locations has been carried out, which reveals the similar trend (Figure 5c). The measured Young’s modulus for HOPG is in the range of the data available in the literature [61,62,63].

During tensile tests, GO and GO/C-SWNT papers showed different mechanical behaviour under in-plane straining. The GO/C-SWNT sample has shown an increment in Young’s modulus by 13% as compared to GO-paper. The intrinsic strength is increased by 126% and the ability to absorb the mechanical energy before rupture is increased by a factor of 5 (see Table 1). This suggests that C-SWNTs are reinforcing more in the in-plane direction than the out-of-plane direction. Though an analogous friction-induced increase in toughness has been observed also in other systems [64,65]. The bridging of C-SWNT through multiple GO sheets effectively transferred the mechanical load, which leads to enhanced toughness for the in-plane direction. The distribution of the nanotubes in the GO sheets are pivotal to drive the mechanical properties. Nevertheless, their aggregation and improper distribution can affect an efficient load distribution with a consequent significant drop in the mechanical properties (see Supplementary Material Figure S10).

In the subsequent stage, GO and the hybrid papers were incorporated into a polystyrene matrix to fabricate PS-GO and PS-GO/C-SWNT laminates and their mechanical behaviour has been investigated through tensile tests. Generally, polymers rich in a large organic phenyl group such as PS are not effectively wettable to the carbon materials like graphene due to the steric hindrance [66,67]. Thus, a hybrid structure abundant in the polar functional group and wettability are suitable to establish with covalent linking of PS. We observed an increment of Young’s modulus and tensile strength for the hybrid fillers in the composite as compared to neat PS and PS-GO laminate. Under tensile loading, the higher shear strength between PS-GO/C-SWNT, interlayer bridging of GO sheets through C-SWNT are responsible for enhanced energy dissipation loading and resulted in higher toughness modulus [68,69,70] than the GO-PS system. Nevertheless, the toughness modulus of the PS is the highest among the PS-GO and PS- GO/C-SWNT due to the lower capability of the carbon materials (graphene and carbon nanotubes) to absorb energy and accommodate large deformation [71].

## 3. Materials and Methods

### 3.1. Synthesis of Free-Standing GO and GO/C-SWNT

Graphene Oxide (GO) and carboxylated single-walled carbon nanotubes (C-SWNTs) were purchased from Graphenea^®^ (Spain) and Cheaptubes (USA), respectively. Commercial graphene oxide dispersion (4 mg/mL) in water was obtained from Graphenea company and was initially ultrasonicated for up to 2 h at 40–50 °C. The elemental analysis of the commercially available GO dispersion is as follows: Carbon (49–56%), Oxygen (41–50%), Nitrogen (0–1), Sulphur (2–4%), Hydrogen (0–1%) 30. Generally, it is easier to disperse GO sheets in DI water within 30 min of sonication. Nevertheless, a higher duration up to 2 h is required both for the homogenous distribution of C-SWNTs in GO-water solution and tailoring the GO flake size for the filtration. The size and the thickness of GO flakes were investigated by Raman and AFM techniques, by depositing a few droplets of the solution on silicon wafer (see Supplementary Material Figure S1). The free-standing paper was obtained by pouring 25 mL of sonicated GO solution over a filtration flask connected to a vacuum pump. A filter paper (Model MF-Millipore, pore size = 0.025 micrometer MCE membrane) has been used for this purpose. After filtration, the deposited GO-paper over filter paper was kept in a fume hood for 24 h at room temperature. As the next step, the GO-papers were dried in the oven for 4 h at 50 °C in air condition. Finally, the free-standing GO-paper was gently removed from the filter paper. The hybrid composite was produced by mixing 25 mg C-SWNT with GO to get 1:4 wt ratio (C-SWNT:GO) dispersion and was sonicated for 2 h. A similar filtration procedure was repeated for the fabrication of the hybrid paper.

### 3.2. Synthesis of Polystyrene-GO and Polystyrene- GO/C-SWNT Laminates

The laminates were prepared by sandwiching GO and GO/C-SWNT free-standing paper between top and bottom of PS (Styron^®^) layers. The polystyrene (PS) was dissolved in chloroform, (i.e., 1 mg/mL) casted in a rectangular Teflon mold and left to dry. Then, GO or GO/C-SWNT (up to 10 mm in length and 5 mm in width) paper was positioned on the top of the PS layer and gently pressed with a metallic roller while raising the temperature to 97 °C for 5 min to promote the adhesion. PS solution was then dropped onto GO and GO/C-SWNT, sandwiching them between the PS layers. The multilayer structure (around 300 mm thick) was allowed to dry for 24 h.

### 3.3. SEM (Scanning Electron Microscopy)

The field emission scanning electron microscopy (FESEM) imaging were carried out at the basal-plains of the GO-paper and to the cross-section area. The fabrication of the cross-section of samples were carried out by inducing fracture in the liquid nitrogen. There was no metallic coating that was sputtered during FESEM imaging. The imaging was taken at a voltage of 5 KV.

### 3.4. Conductivity and CV (Cyclic Voltammetry) Tests

Conductivity measurement was performed in a Keithley 2420 source meter by applying a linear voltage and monitoring the current across the paper. The free-standing GO sheets and its C-SWNT hybrid paper were cut into rectangular sheets. A conductive silver paint was deposited at the end of the sheets, the Keithley probes were placed between the conductive electrodes which were 2 cm apart. The electrical conductivity of the samples were monitored at room temperature by applying a sweeping DC electric voltage from −20 V to +20 V. Electrochemistry was performed in a 3-electrode cell with films as the working electrode, Pt as the counter electrode and Ag/AgCl as the reference electrode in PBS (Phosphate-buffered saline) = 7.

### 3.5. XRD (X-ray Diffraction)

X-ray diffraction patterns were recorded on a APD 2000 X-ray diffractometer with a Cu K_α_ radiation (λ = 1.540598 Å) equipped with a NaI (Tl) scintillation detector and a Goebel Mirror optics. Radiation was generated from a Cu anode supplied with 40 keV and a current of 30 mA. The diffraction angle scan range for 2θ was 5° to 30° with a step size of 0.02° at a rate of 5 s/step.

### 3.6. XPS (X-ray Photoelectron Spectroscopy)

XPS analyses were performed using an Axis DLD Ultra from Kratos (UK). Wide survey spectra in low energy resolution conditions were acquired on both GO- and C-SWNT/GO-paper samples. For these spectra the pass energy was set to 150 eV. High-resolution core lines were then acquired at a pass energy of 20 eV to enable assignment of chemical bonds. With this aim, core lines were fitted by using a homemade software based on the R platform (for additional information, see the R-project website https://www.r-project.org). A linear background subtraction was performed for all the core lines. An asymmetric Voigt function was utilised for the graphitic component of the C1s while for all the other components a Gaussian profile was used.

### 3.7. TGA (Thermogravimetric Analysis)

Thermal stability of GO and GO + C-SWNT paper were characterised using thermogravimetric analysis (TGA) utilising TGA/DSC 2 instrument from Mettler Toledo. A small piece of sample was cut from GO and GO + C-SWNT paper and put in an alumina crucible. After transferring into the instrument, the sample temperature was then increased from room temperature to 800 °C at a ramp of 10 °C min^−1^ under an Ar flow of 50 mL min^−1^. Mass change and heat flow during the increase of sample temperature were recorded using the STAR^e^ system.

### 3.8. Tensile and Nanoindentation Tests

In order to derive the mechanical behavior of GO and GO+C-SWNT composite materials, rectangular samples were cut and tested through a tensile testing machine. The samples were about 11 mm long and 4 mm wide. Repeated Tests up to 3 times were conducted at room temperature at a speed of 0.05 mm/s (corresponding to 0.4% s^−1^) using MIDI 10 by Messphysik Materials Testing. The nanoindentation measurements were carried out through force-distance spectroscopy using cantilever (Model: DTNCHR, Resonance frequency: 340KHz, stiffness (from Sader’s Method): 75 ± 10 N/m) and its apex was coated with diamond-like carbon (DLC). The DLC coating avoids the wear of the tip apex but results in a broader diameter (≈250–300 nm) as confirmed from the SEM image. The indentation were carried out more than 15 times over GO-paper, GO+C-SWNT paper and commercially available HOPG was used as a reference.

### 3.9. AFM Conductivity and Kelvin Probe Force Microscopy (KPFM)

Conductive AFM (C-AFM) and KPFM measurements were carried out on AFM instrument from Bruker (Model: Dimension, icon, ScanAsyst) in ambient conditions at 33% relative humidity. Drop casted C-SWNT on GO film was prepared for the investigation. Cantilever (PFTUNA (Pt/Ir coating) and PFQNE-AL) from Bruker, Santabarbara (USA) were used for the investigation of conductive mapping and surface potential measurements. The resonance frequency of the cantilevers were recorded as 73 KHz and 342 KHz and Kn = 0.4 and 0.8 N/m with 20% of deviation during “thermal tuning” method. During the C-AFM measurement, DC bias of 900 mV was applied between the probe and the sample. The current (nA) passing through the sample is recorded under an effective contact condition named as contact current (a duration between jump-to-contact and pull-out). The intensity of the current in the C-AFM is attributed to the current flowing between the probe apex of a conductive tip (coating of Pt/Ir) and the sample. The contact area between the tip-sample results in a spreading resistance and the local resistivity to the test sample. The significant contrast appears in the GO/C-SWNT hybrid composite distinguishes the local electrical properties of the 1D and 2D materials and its associated functional group.

The KPFM measurement was carried out in Peak force-KPFM mode in the two-pass techniques. In the second pass, lift-off height of the tip was 10 nm. The advantage of using this technique is to remove image artefacts induced by topography, thereby increasing the resolution and accuracy of the electrical measurement. The work function of the probe was measured using a freshly cleaved highly oriented pyrolytic graphite (HOPG) surface at lift height of ~10 nm, which is calculated as 4.2 eV. The work function of the freshly cleaved HOPG was taken as 4.6 eV. No external bias was carried out during CPD measurement.

### 3.10. Micro-Raman Analysis

Raman spectra were acquired with LabRAM Aramis (HORIBA Jobin Yvon, Lille, France), the excitation wavelength used was 632.8 nm generated from an He-Ne laser having an output power of approximately 15mW. The diffraction grating used had 1200 lines cm^−1^ and the CCD detector Peltier-cooled was at ~−68 °C. The acquisition range for each spectrum was 1200 to 3200 cm^−1^ and each spectrum was computed by averaging two successive integrations of 10 s each. The objective lens of the microscope (Olympus BX41 from Olympus America Inc., Center Valley, PA, USA) used was a 100× with a long working distance. Aperture slit and pinhole were set respectively to 1000 µm and 100 µm. All the Raman spectra are treated with base line subtraction and are normalized to G peak. The Raman spectra of individual GO sheets and SWNT prior to the fabrication of free-standing paper are illustrated at Supplementary Material Figure S2.

### 3.11. Drop Shape Contact Angle Measurement

The wettability of GO and GO/C-SWNT paper surfaces were evaluated through drop shaped contact angle measurements utilizing an FTA 1000 Analyzer System. The contact angle was measured through deionized water drops (volume 20 µL) automatically deposited on the sample surface.

## 4. Conclusions

We report a wet-chemical method to fabricate a free-standing GO/C-SWNT hybrid paper from the aqueous dispersion of GO sheets and functionalised SWNTs. The addition of nanotubes in GO platelets has been demonstrated as a method to enable a combination of multiple properties, including electrical, mechanical, and thermal stability. The presence of C-SWNTs between GO sheets is useful to establish conductive pathways for charge mobility. Even a single C-SWNT can modify the local surface potential at the GO surface as reflected by the CPD contrast, which increases with the network of the C-SWNTs. The capacitance is increased up to 3.5 times in the hybrid structure. The thermal degradation changed dramatically by incorporating 25% of C-SWNT and the mass loss is reduced. The mechanical performance of the hybrid structure is limited to in-plane straining; the nanoindentation-induced out-of-plane stress showed a lower Young’s modulus of GO/C-SWNT than the GO-paper and the reference HOPG. The tensile measurement displayed the superior performance of the hybrid structure due to the establishment of the interaction between the GO sheets by C-SWNT. These results suggest a way of designing advanced composites with superior mechanical properties by incorporating two nanomaterials such as graphene oxide and carbon nanotube. Several applications can be explored using the developed hybrid/polymer systems from charge storage, tailored mechanical properties and tuned conductivity.

## Figures and Tables

**Table 1:** Nanoindentation and tensile tests of GO, GO/C-SWNT and PS composites.

Sample in Nanoindentation Tests	Equation (2) Fit		
	*E* [GPa]	*γ* [N/m]	Average R^2^
GO	1.47 ± 0.70	0.56 ± 0.73	0.99
GO/C-SWNT	0.23 ± 0.12	negligible	0.91
HOPG	11.41 ± 1.89	0.21 ± 0.50	0.92
**Sample in Tensile Tests**	**Young’s Modulus**	**Tensile Strength**	**Toughness Modulus**
	**[MPa]**	**[MPa]**	**[KPa]**
GO	652 ± 127	3.4 ± 0.4	9 ± 1
GO/C-SWNT	738 ± 76	7.7 ± 1.2	50 ± 20
PS (neat)	153 ± 42	3.1 ± 1.1	150 ± 50
PS-GO	178 ± 9	2 ± 0.3	12 ± 1
PS-GO/C-SWNT	294 ± 77	5.5 ± 2	100 ± 30

## Supplementary Materials

The following are available online at https://www.mdpi.com/1422-0067/21/22/8585/s1.
