# Supplementary material for: Free-Standing Graphene Oxide and Carbon Nanotube Hybrid Papers with Enhanced Electrical and Mechanical Performance and Their Synergy in Polymer Laminates
Source: Int J Mol Sci. 2020 Nov 14;21(22):8585. doi: 10.3390/ijms21228585 (PMC7696645; doi:10.3390/ijms21228585)
Supplement: Supplementary file 1 [file ijms-21-08585-s001.pdf]

International Journal of
Molecular Sciences

MDPI

Supplementary Materials

Free-Standing Graphene Oxide and Carbon Nanotube Hybrid Papers with Enhanced Electrical and Mechanical Performance and Their Synergy in Polymer Laminates

Manoj Tripathi [1,*], Luca Valentini [2], Yuanyang Rong [1], Silvia Bittolo Bon [2], Maria F. Pantano [3], Giorgio Speranza [4,5,6], Roberto Guarino [3,†], David Novel [3,4], Erica Iacob [4], Wei Liu [4], Victor Micheli [4], Alan B. Dalton [1] and Nicola M. Pugno [3,7,*]

1 Department of Mathematics and Physical Sciences, University of Sussex, BN1 9QH Brighton, UK; tracy.rong0202@gmail.com (Y.R.); A.B.Dalton@sussex.ac.uk (A.B.D.)
2 Civil and Environmental Engineering Department, University of Perugia, INSTM Research Unit, Strada di Pentima 4, 05100 Terni, Italy; luca.valentini@unipg.it (L.V.); silvia.bittolobon@unipg.it (S.B.B.)
3 Laboratory of Bio-inspired, Bionic, Nano, Meta Materials & Mechanics, Department of Civil, Environmental and Mechanical Engineering, University of Trento, via Mesiano 77, 38123 Trento, Italy; maria.pantano@unitn.it (M.F.P.); roberto.guarino@alumni.unitn.it (R.G.); ddsnovel@gmail.com (D.N.)
4 Centre for Materials and Microsystems, Fondazione Bruno Kessler, via Sommarive 18, 38123 Trento, Italy; g.speranza@fbk.eu (G.S.); iacob@fbk.eu (E.I.); liuwei4176@hotmail.com (W.L.); micheli@fbk.eu (V.M.)
5 Department of Industrial Engineering, University of Trento, via Sommarive 9, 38123 Trento, Italy
6 Istituto di Fotonica e Nanotecnologie, IFN-CNR, via alla Cascata 56/C, 38123 Trento, Italy
7 School of Engineering and Materials Science, Queen Mary University of London, Mile End Road, London E1 4NS, UK
* Correspondence: m.tripathi@sussex.ac.uk (M.T.); nicola.pugno@unitn.it (N.M.P.)
† Present address: École Polytechnique Fédérale de Lausanne (EPFL), Swiss Plasma Center (SPC), CH-5232 Villigen PSI, Switzerland.

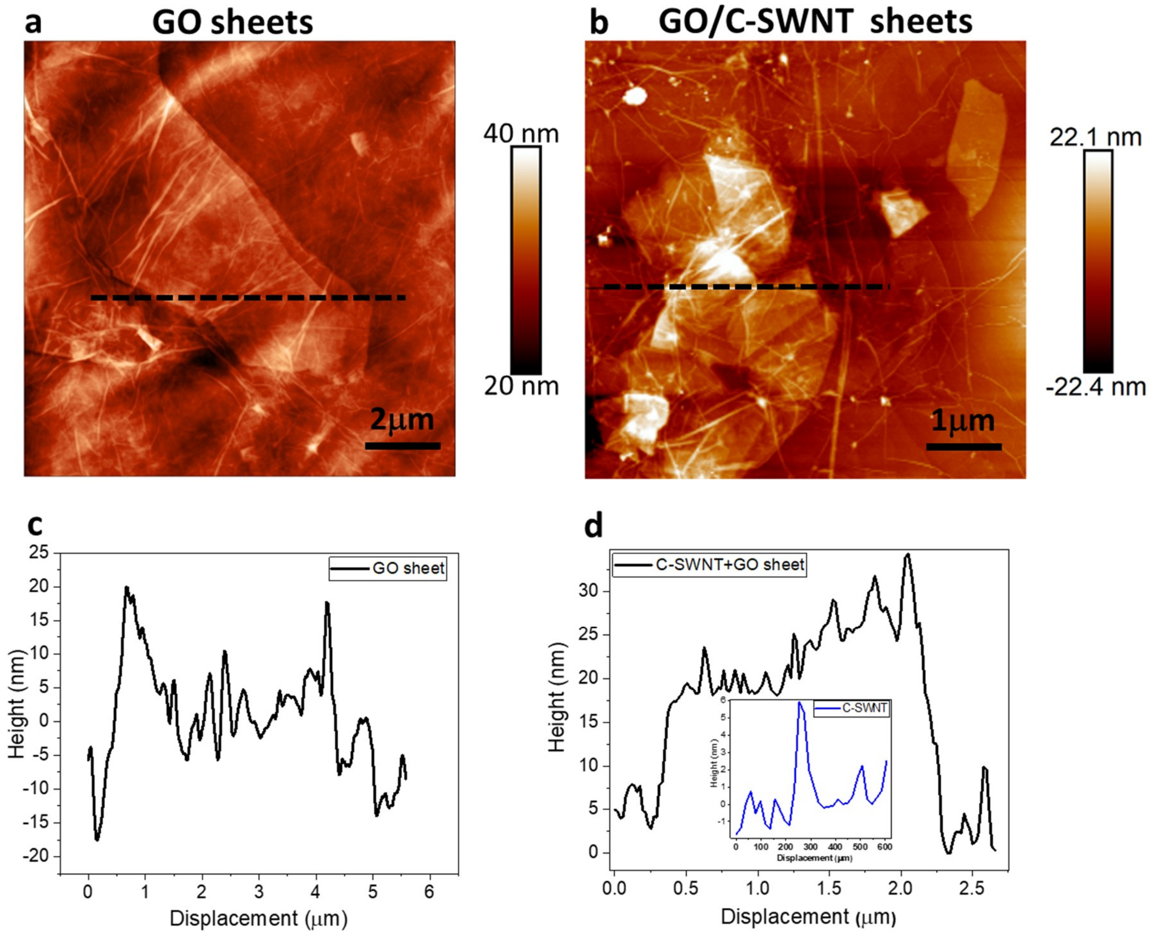

Figure S1. (**a**) AFM topography image (10 × 10 μm²) of GO and (5 × 5 μm²) for (**b**) C-SWNT-GO sheets after 2 h of sonication. The dispersion of GO flakes is drop cast over Si wafer and dried in ambient condition. (**c**)The AFM profile reveals thickness (between 20–40 nm) and lateral flake size of GO sheet up to 10 microns. (**d**) The line profile of C-SWNT-GO sheets indicates the comparable thickness of GO sheets, where each C-SWNT are connected with different GO sheets. The individual thickness of a single C-SWNT is presented in the inset.

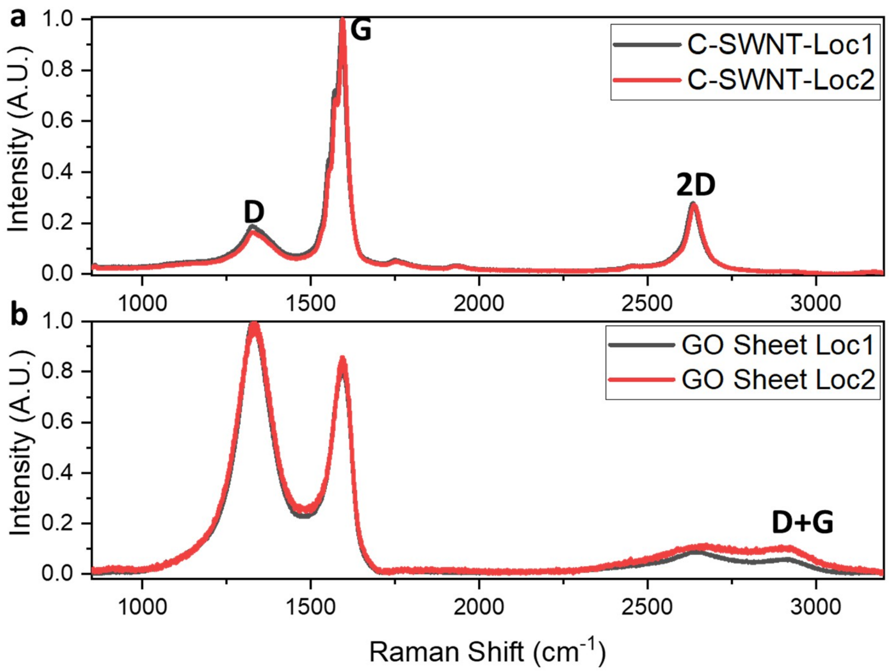

Figure S2. Raman spectra of C-SWNT and GO sheets produced after 2hrs of sonication at two different locations (Loc1 and Loc2). Both spectra shows typical peak of G, D, 2D and D+G Raman modes [1,2].

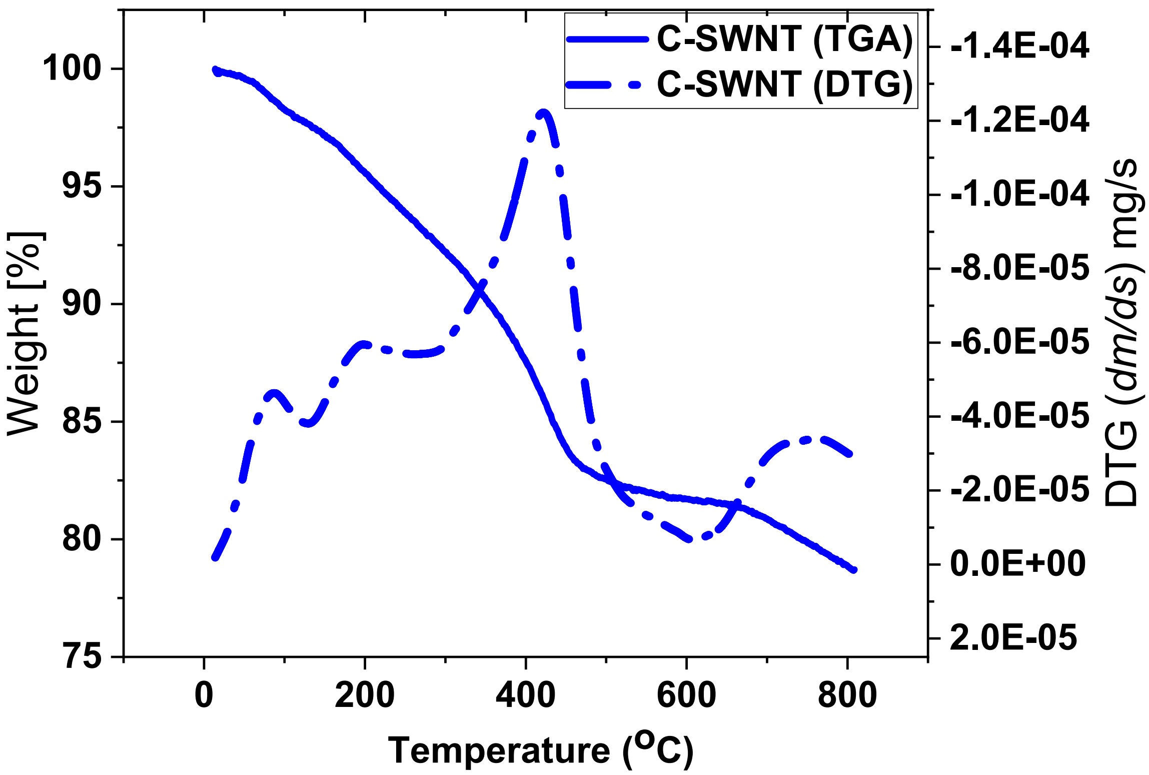

Figure S3. TGA and DTG of SWNT. The TGA curve shows only about 4 wt% mass loss, which is much less than that of GO and GO/CNT paper. This is because on the CNT surface there is less functional groups than GO. The majority of mass loss occurred between 320–480 ^{o}C for SWNT.

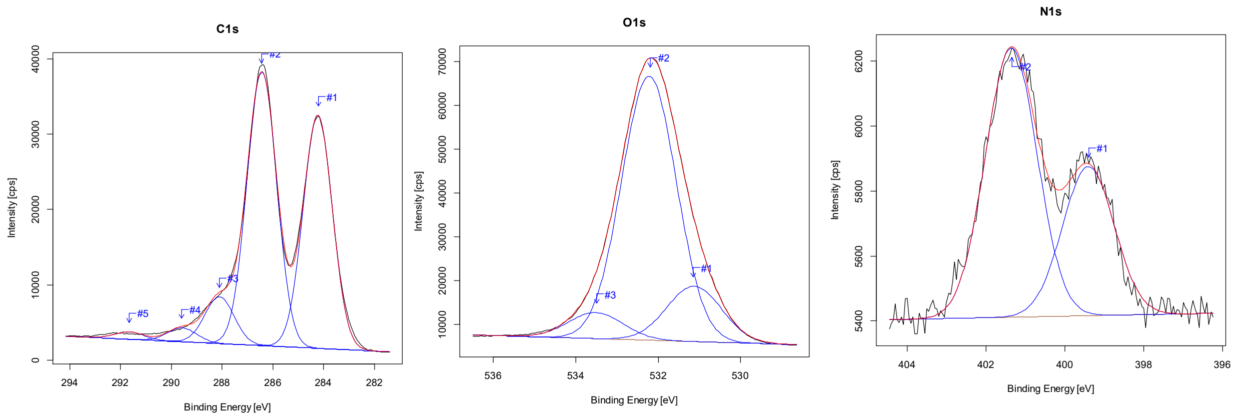

Figure S4. XPS details of GO.

Table S1. GO paper.

	Components	Area(cps)	FWHM	RSF	Be (eV)	TOT.(%)
	C1s	7900928.36		0.278		68.56
#1	C1	3206166.88	1.35	0.278	284.22	27.82
#2	C2	3756505.88	1.35	0.278	286.43	32.60
#3	C3	644820.08	1.35	0.278	288.10	5.60
#4	C4	191873.09	1.35	0.278	289.59	1.66
#5	C5	101562.44	1.35	0.278	291.65	0.88
	O1s	3352965.37		0.780		29.09
#1	C1	536256.20	1.56	0.780	531.13	4.65
#2	C2	2564524.36	1.56	0.780	532.22	22.25
#3	C3	252184.81	1.56	0.780	533.52	2.19
	N1s	88280.60		0.477		0.77
#1	C1	31462.77	1.54	0.477	399.41	0.27
#2	C2	56817.83	1.54	0.477	401.36	0.49

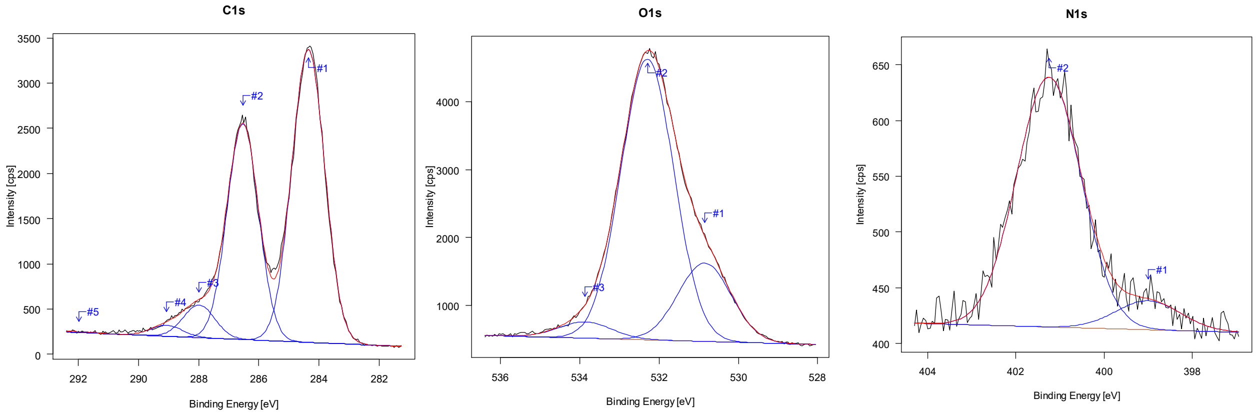

Figure S5. XPS details of GO/C-SWNT paper.

Table S2. GO/C-SWNT paper.

	Components	Area(cps)	FWHM	RSF	Be (eV)	TOT.(%)
	C1s	8172.49			284.296	69.04
#1	C1	4423.4	1.28	0.278	284.35	37.37
#2	C2	3108.20	1.22	0.278	286.53	26.26
#3	C3	471.82	1.22	0.278	287.99	3.99
#4	C4	158.97	1.22	0.278	289.07	1.34
#5	C5	10.08	1.22	0.278	291.98	0.09
	O1s	9231.82			532.246	27.80
#1	C1	1925.11	1.57	0.780	530.85	5.80
#2	C2	6907.66	1.57	0.780	532.30	20.80
#3	C3	399.04	1.57	0.780	533.87	1.20
	N1s	458.42			401.296	2.26
#1	C1	47.56	1.72	0.477	399.00	0.23
#2	C2	410.86	1.72	0.477	401.25	2.02

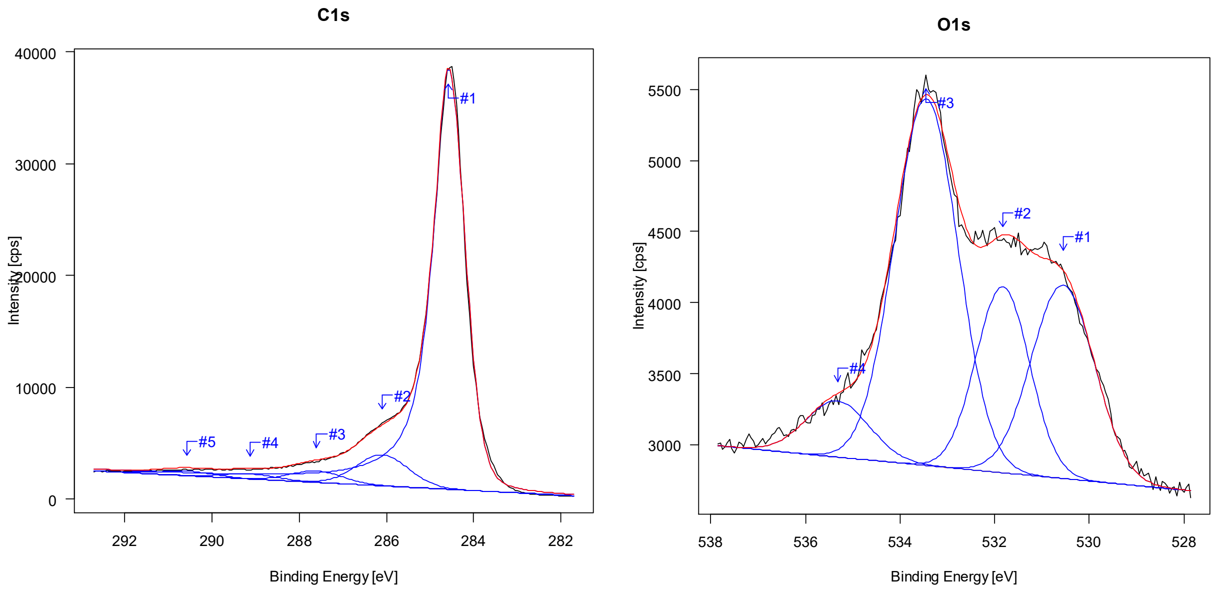

Figure S6. XPS details of GO after TGA.

Table S3. GO paper after TGA.

	Components	Area(cps)	FWHM	RSF	Be (eV)	TOT.(%)
	C1s	50109.08			284.500	93.51
#1	C1	43168.25	0.35	0.278	284.59	80.55
#2	C2	4124.44	1.40	0.278	286.09	7.70
#3	C3	1520.66	1.40	0.278	287.60	2.84
#4	C4	677.93	1.40	0.278	289.12	1.27
#5	C5	617.80	1.40	0.278	290.58	1.15
	O1s	9146.14	1.58		533.450	93.51
#1	C1	2285.14	1.38	0.78	530.56	1.52
#2	C2	1858.50	1.38	0.78	531.83	1.24
#3	C3	4339.27	1.38	0.78	533.45	2.89
#4	C4	663.23	1.38	0.78	535.32	0.44

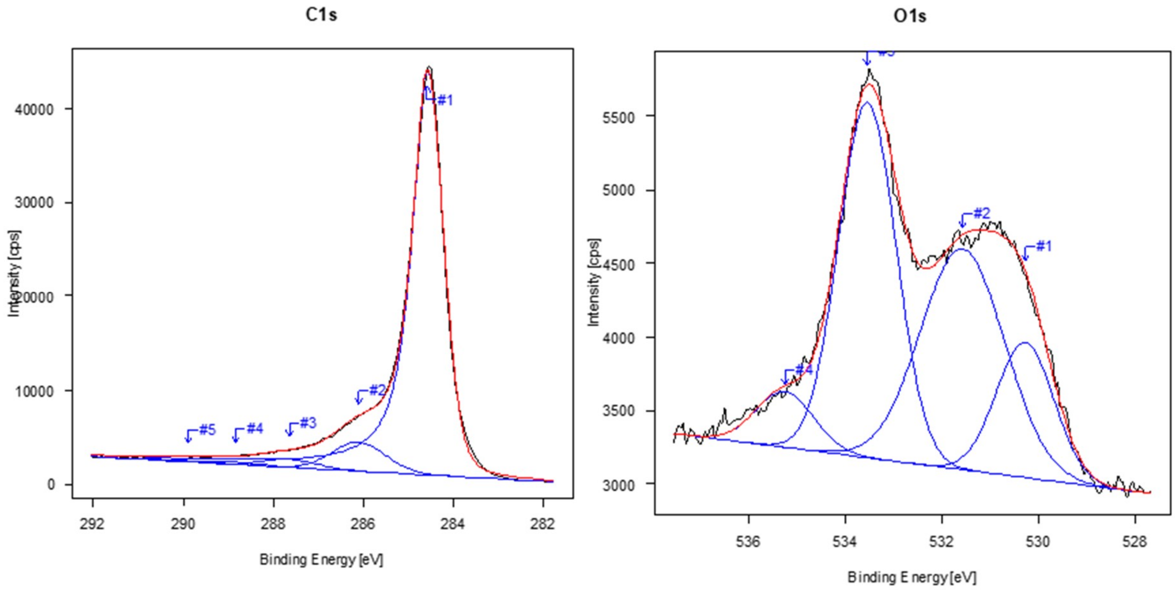

Figure S7. XPS details of GO/C-SWNT after TGA.

Table S4. XPS details of GO/C-SWNT after TGA.

	Components	Area(cps)	FWHM	RSF	Be (eV)	TOT.(%)
	C1s	53731.06			284.550	94.08
#1	C1	47380.41	0.33	0.278	284.59	82.96
#2	C2	4565.83	1.40	0.278	286.10	7.99
#3	C3	1253.85	1.40	0.278	287.64	2.20
#4	C4	338.36	1.40	0.278	288.84	0.59
#5	C5	192.62	1.40	0.278	289.89	0.34
	O1s	8819.18			533.500	5.50
#1	C1	1369.46	1.40	0.780	530.27	0.85
#2	C2	3273.71	2.05	0.780	531.57	2.04
#3	C3	3610.30	1.40	0.780	533.54	2.25
#4	C4	565.71	1.40	0.780	535.25	0.35

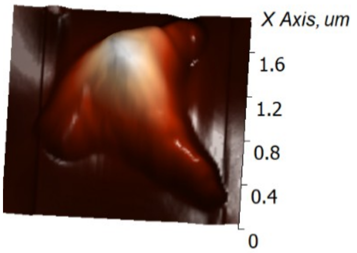

a

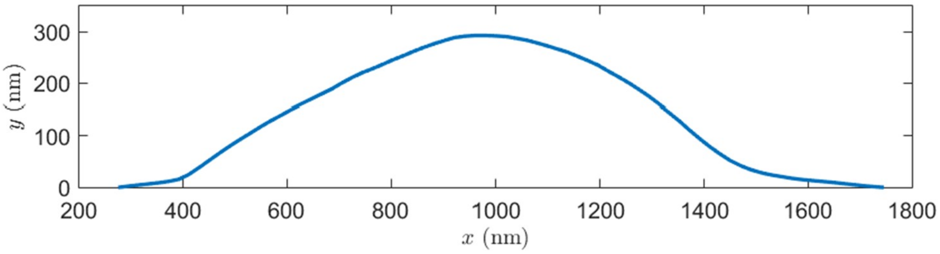

b

Figure S8. (**a**) Tip apex characterizations, 3D AFM image of a convoluted tip obtained by scanning over grating sample (Model: TGT1, NT-MDT) and (**b**) line profile of the pyramidal AFM tip geometry.

Supplementary Information 1: Analysis of the nanoindentation curves.

The multilayer GO, GO+C-SWNT and HOPG samples are considered as a homogeneous material, since all the layers are supposed to have the same composition and thus elastic properties, while the tip is considered rigid.

Assuming that the base triangle is equilateral, denoted with α is the pyramid angle, the projected contact area is given by:

$$A = 3\sqrt{3}\, h_c^2\ \tan^2 \alpha \tag{S1}$$

being h_c the contact depth. Therefore, according to [3], the contact stiffness becomes:

$$S = 2\,E^* \sqrt{\frac{A}{\pi}} = 2\,E^* \sqrt{\frac{3\sqrt{3}}{\pi}}\, h_c \tan\alpha \tag{S2}$$

where $E^* \approx {E}/{(1-\nu^2)}$, with E the Young's modulus and ν the Poisson's ratio of the substrate.

By inserting the indentation depth $h = {\pi\, h_c}/{2}$ (from [3]), and integrating Equation (S2), we obtain the elastic force during nanoindentation:

$$F_{el} = \int S\mathrm{d}h = \frac{2}{\pi}\sqrt{\frac{3\sqrt{3}}{\pi}}\,\frac{E}{1-\nu^2}\tan\alpha\ h^2 \tag{S3}$$

To take into account the adhesive contributions, it is necessary to consider the tip-sample contact surface, which in our case is given by:

$$A_c = 3\sqrt{3}\,\frac{\tan\alpha}{\cos\alpha}\,h_c^2 \tag{S4}$$

Again following [5], and introducing the indentation depth h, the adhesive force is given by:

$$F_{ad} = -\gamma\frac{\mathrm{d}A_c}{\mathrm{d}h} = -\gamma\frac{24\sqrt{3}}{\pi^2}\,\frac{\tan\alpha}{\cos\alpha}\,h \tag{S5}$$

where γ is the adhesion energy.

By summing Equations (3) and (5), the experimental nanoindentation data can be fitted by a curve of the type:

$$F = \frac{2}{\pi}\sqrt{\frac{3\sqrt{3}}{\pi}}\,\frac{E}{1-\nu^2}\tan\alpha\ h^2 - \gamma\frac{24\sqrt{3}}{\pi^2}\,\frac{\tan\alpha}{\cos\alpha}\,h \tag{S6}$$

The force-depth curves are fitted up to a penetration depth of 40 nm for GO and GO+C-SWNT, while up to 10 nm for HOPG. This choice derives from the need of considering only the elastic part of the nanoindentation curves, thus avoiding effects at large displacements (e.g. plasticity, sliding of the interfaces, etc.) that are not accounted for by the theoretical analysis discussed here. According to the discussion in the main text, the tip geometry can be described by a pyramid angle $\alpha \approx 70°$. In addition, we use $\nu \approx -0.2$ as Poisson's ratio of graphene oxide samples, following the results of Wan *et al.* [4] and considering the degree of oxidation of the present GO samples, and $\nu = 0.16$ for HOPG [3].

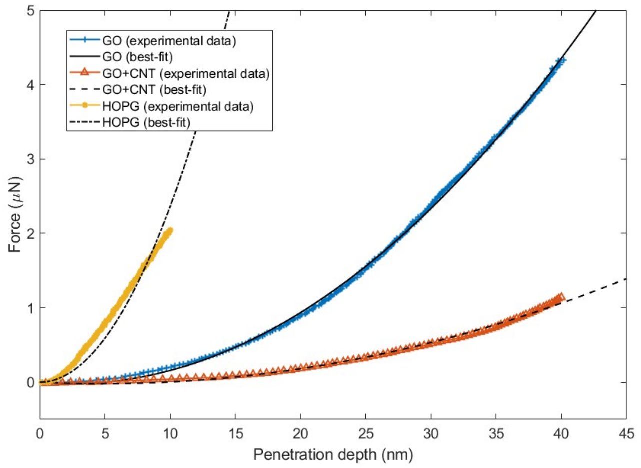

Figure S9. Example fits through Equation (S6) of the nanoindentation curves of GO, GO+C-SWNT and HOPG.

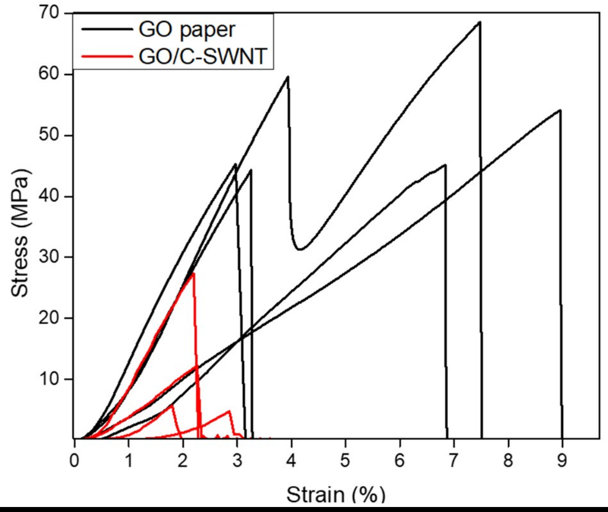

[redacted]		
[redacted]	46.3 ± 11	7.9 ± 8.1
[redacted]	5.6% ± 2.2%	3.2% ± 2.3%
[redacted]	1.32 ± 0.44	0.63 ± 0.49

Figure S10. Tensile measurement of free-standing GO and GO/C-SWNT paper. Here the nanocomposites are not produced by magnetic stirring, which leads to the aggregation of the CNT. The improper distribution of the C-SWNTs in the GO is unable to form bridging sites and causes accumulation of tensile strain. Thus, the hybrid material has a decrease in strength and Young's modulus as compared to its native GO of similar thickness.

References

[1] M. V. O.; Lombardo, A.; Kulmala, T. S.; Ferrari, A. C., Quantifying Defects in Graphene via Raman Spectroscopy at Different Excitation Energies. Nano Letters 2011, 11 (8), 3190-3196.

[2] Raman spectroscopy of carbon nanotubes M.S.Dresselhausa G.DresselhausbR.SaitocA.Jorio Physics Reports Volume 409, Issue 2, 2005, Pages 47-99.

[3] L. Sirghi, J. Ponti, F. Broggi, F. Rossi, Probing elasticity and adhesion of live cells by atomic force microscopy indentation, European Biophysics Journal 37(6) (2008) 935-945.
[4] J. Wan, J.-W. Jiang, H.S. Park, Negative Poisson's ratio in graphene oxide, Nanoscale 9(11) (2017) 4007-4012.
[5] O. Blakslee, D. Proctor, E. Seldin, G. Spence, T. Weng, Elastic constants of compression-annealed pyrolytic graphite, Journal of Applied Physics 41(8) (1970) 3373-3382.
